# Improvements at Charing Cross Hospital

**Published:** 1903-10-10

**Authors:** 


### IMPROVEMENTS AT CHARING CROSS HOSPITAL.

The extensive alterations now in progress at Charing Cross Hospital will, it is hoped, be fairly completed early in 1904. They comprise, in addition to the nursing home on the Chandos Street side, rooms for the resident staff, new surgical and special wards, and new out-patients' and casualty departments. The imposing stone staircase facing the main entrance has been removed, thus making space available for a larger double entrance-hall and rooms for the honorary staff, while access to the upper floors is effected by means of two large "Otis" elevators, which work automatically, and by a staircase enclosing the well in which they are placed. On the entrance floor is also a waiting-room for visitors, etc., an assistant secretary's room, and the residents' dining-room, which has been redecorated. The residents' bed and sitting-rooms are in the new part of the building, and are not yet sufficiently advanced for inspection. They are approached by a corridor from the entrance-hall. On the first, second, third, and fourth floors of the new portion—facing King William Street—are the new surgical wards, male and female, with sisters' rooms, small "quiet" wards, and operating theatres. The wards are still in the hands of the plasterers, but it is evident that they are large and lofty, with good lights and cross ventilation, the triangular space in the centre of the block being left unbuilt upon. The old and new portions are connected by bridges with the Nursing Home, and these form a means of escape from the wards in case of fire. An isolation tower for suspected cases has been added to the old block; this is reached by spiral iron staircases, and by a patients' lift. There are two balconies, and the air at this altitude is extremely fresh. The plan of isolated sinks, situated at the ends of these balconies, has been adopted. A new Jewish ward has been built, and the five beds endowed by members of the Levi family have been transferred to it. Adjoining are kitchens for the preparation of special food for Hebrew patients. The ward is a light and cheerful one, coloured terra-cotta and cream; the windows being of the Louvre pattern, and the ventilators those known as the "Inlet," with hopper and outside grating. These have also been placed in the old wards, to each of which two new windows have been added, together with a door leading to the fire-escape exit.

The new out-patients' department is situated in the basement of the King William Street block, and the well between that block and the Nurses' Home, the entrance being in King William Street. Kitchens, linen-receiving rooms, and other domestic offices, also occupy this floor, and there is a sub-basement devoted to medical baths. Masaic and terrazo are being used for landings, and opaline tiles for passages, those on the main staircases being white and pale green. The number of beds added to the hospital will be 83, bringing up the total to 243.

The architect is Mr. A. Saxon Snell, F.R.I.B.A., and the builders are Messrs. Holloway.

